# A label-free nanowell-based impedance sensor for ten-minute SARS-CoV-2 detection†

**DOI:** 10.1039/d5sd00002e

**Published:** 2025-04-30

**Authors:** Zhuolun Meng, Liam White, Pengfei Xie, Hassan Raji, S. Reza Mahmoodi, Aris Karapiperis, Hao Lin, German Drazer, Mehdi Javanmard, Edward P. DeMauro

**Affiliations:** a Department of Electrical and Computer Engineering, Rutgers University-New Brunswick Piscataway NJ 08854 USA mehdi.javanmard@rutgers.edu; b Department of Mechanical and Aerospace Engineering, Rutgers University-New Brunswick Piscataway NJ 08854 USA edward.demauro@rutgers.edu

## Abstract

This work explores label-free biosensing as an effective method for biomolecular analysis, ensuring the preservation of native conformation and biological activity. The focus is on a novel electronic biosensing platform utilizing micro-fabricated nanowell-based impedance sensors, offering rapid, point-of-care diagnosis for SARS-CoV-2 (COVID-19) detection. The nanowell sensor, constructed on a silica substrate through a series of microfabrication processes including deposition, patterning, and etching, features a 5 × 5 well array functionalized with antibodies. Real-time impedance changes within the nanowell array enable diagnostic results within ten minutes using small sample volumes (<5 μL). The research includes assays for SARS-CoV-2 spike proteins in phosphate-buffered saline (PBS) and artificial saliva buffers to mimic real human SARS-CoV-2 samples, covering a wide range of concentrations. The sensor exhibits a detection limit of 0.2 ng mL^−1^ (1.5 pM) for spike proteins. Middle East respiratory syndrome (MERS-CoV) spike proteins are differentiated from SARS-CoV-2 spike proteins, demonstrating specificity.

## Abstract 2

This work explores label-free biosensing for rapid COVID-19 detections. Specifically, a novel electronic biosensing platform utilizing microfabricated nanowell-based impedance sensors.

## Graphical abstract

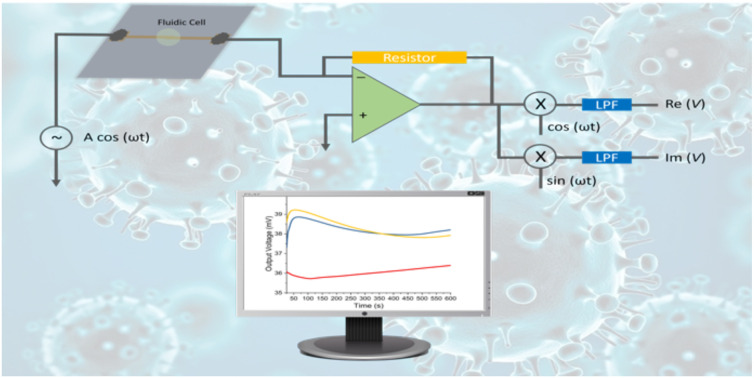

## 1 Introduction

Coronavirus disease 2019 (COVID-19) is an infectious contagious disease caused by the SARS-CoV-2 virus. In December 2019, the first known case of COVID-19 was identified in Wuhan, China.^1^ In the following months, the SARS-CoV-2 virus rapidly spread across the world and now has multiple mutations.^2–4^ According to the World Health Organization (WHO) data, the cumulative total reported cases by Mar 2, 2025, was 777 594 331.^5^ COVID-19 significantly impacted the global economy, food security, education, and mental health, among other effects.^6^

There are ongoing efforts to fight against respiratory diseases with high transmissibility by researchers and scientists from different disciplines.^4,6–8^ The development of vaccines and treatment strategies has successfully decreased hospitalization and mortality rates.^9–11^ In addition, to potentially control the spread of the disease, detection is the first line of defense and is one of the successful responses to the pandemic.^8,12^ Testing is also pivotal for public health, and detection of SARS-CoV-2 helps investigators characterize its prevalence, spread, and contagiousness.^8^

There are multiple diagnostics methods developed for COVID 19 in the past few years, which can be categorized into RNA & DNA/molecular diagnosis, antibody/antigen testing, clinical imaging techniques, and biosensors.^13–16^ RNA & DNA/molecular diagnosis are some of the most developed detection methods.^14,15^ They are highly sensitive, accurate, and specific for SARS-CoV-2 virus detection.^14,15^ However, these methods have disadvantages, including the need for trained operators and long workflow times (from 30 minutes to several days).^14,15^ Antibody/antigen testing methods have specific advantages, including large capacity, rapid results, inexpensive materials, portability, and ease of operation.^13–15^ These methods, however, are not as accurate as molecular diagnosis.^13–15^ Other detection methods are based on medical imaging techniques, especially computed tomography (CT), X-ray imaging, and ultrasound, which analyze chest images to diagnose patients. These detection methods are non-invasive and could be implemented for fast screening, especially in combination with automated image analysis.^14,15,17^ However, the equipment cost and radiation exposure need to be taken into consideration, particularly in the case of CT scans, which expose patients to significant amounts of radiation and cannot be used frequently.^14,15,17^ As the technologies develop, biosensors are becoming a reliable option for disease detection and diagnosis. Compared to the detection methods discussed above, biosensor-based methods present alternatives that do not need advanced equipment and skilled operators for rapid diagnosis.^15,18–21^ In particular, label-free devices for bio-detection have developed significantly over the last decade. Detection utilizing label-free devices of biomarkers has numerous advantages compared to label-based counterparts, including cost-effectiveness, simpler sample preparation, a broad range of targets, portability, and point-of-care capabilities.^22–24^

In this study, a microfabricated label-free nanowell array impedance sensor is used to detect SARS-CoV-2 spike proteins in artificial saliva. In previous research, this sensor was used to detect stress hormones and cytokine in serum.^25–30^ A new preparation and data analysis method for a nanowell sensor is presented which demonstrates a lower limit of detection (LOD). Additionally, the sensor can discriminate between SARS-CoV-2 spike proteins and MERS-CoV spike proteins, demonstrating target specificity.

## 2 Materials and methodology

### 2.1 Impedance sensor methodology

**Fig. 1:**
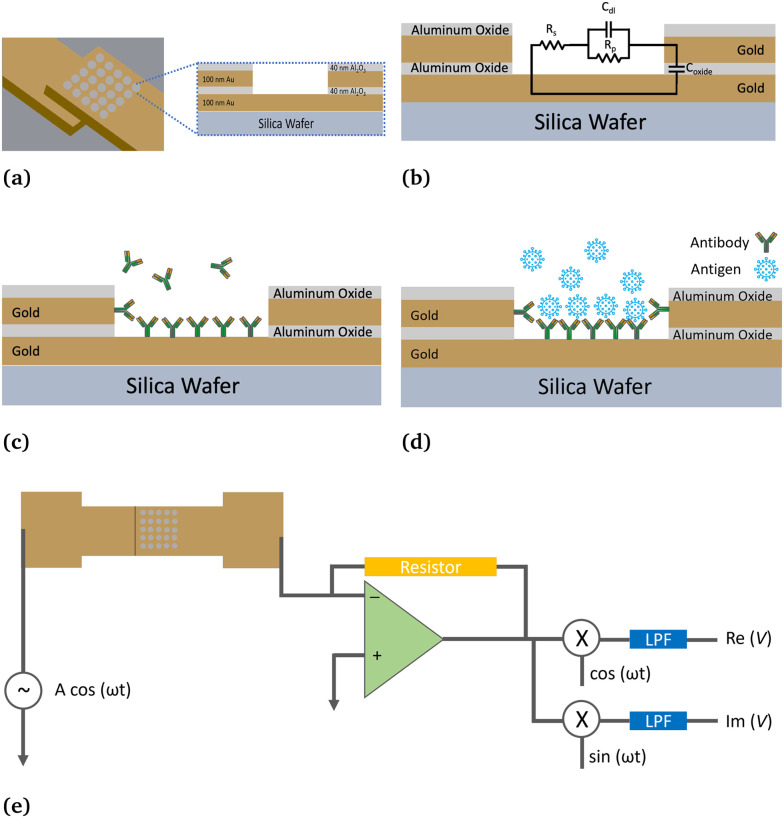
(a) Cross-sectional view of a single nanowell. (b) Equivalent circuit model. (c) Cross-sectional view of single nanowell adsorbing antibodies. (d) Cross-sectional view of the nanowell adsorbing target proteins. (e) Equivalent circuit of measurement platform using a lock-in amplifier.

The sensor is a 5 × 5 array of wells microfabricated over a 20 μm × 20 μm area consisting of two opposing gold electrodes separated by an aluminum oxide layer. Antibodies are first injected and adsorbed in the wells. A sample is then introduced, and the impedance between electrodes is monitored through lock-in amplifiers (operational frequency: 1 MHz, analog bandwidth: 0.7 μHz–50 MHz, sensitivity: 1 nV to 1.5 V, size: 45 × 35 × 10 cm, gain: 1000, sampling rate: 225 s^−1^, HF2IS 50 MHz Lock-in Amplifier, Zurich Instruments AG, Technopark Strasse 1, 8005 Zurich, Switzerland) to determine the presence of the corresponding antigen. The schematic cross-section view of a single well in the array is shown in Fig. 1a, indicating the two gold layers acting as electrodes and separated by a thin dielectric layer of aluminum oxide. The equivalent circuit model is shown in Fig. 1b, and discussed in detail in previous research by Mahmoodi *et al.*^27^

The following is a brief description of the testing procedure. First, PBS buffer is injected inside the wells to create a liquid environment. Then, the selected antibodies are injected into the wells and adsorbed on their surface. The changes in impedance between the two gold electrodes are monitored in real-time using a lock-in amplifier to determine if the antibodies adsorbed successfully, as shown schematically in Fig. 1c (see a top view schematic in Fig. S1a in the ESI† material). Subsequently, the test sample is introduced into the wells, and if there is any adsorptions happen, the changes in impedance are continuously monitored to detect the binding of antigens onto the antibodies (see schematic of the binding in Fig. 1d and the corresponding top view schematic in Fig. S1b in the ESI† material). The biosensor is applied 100 mV voltage at 1 MHz operational frequency. Measurable increases in impedance indicate the presence of antigen–antibody pairs after introducing the test sample. Although the impedance changes are measured by measuring the current signals, the first stage of the lock-in amplifier is a mixer that compares the reference voltage with the signal measured. Thus, we need to convert the signal to voltage to align with the reference voltage for the lock-in amplifier to process the data further. The circuit topology is Fig. 1e, which shows the Zurich Instruments Current Amplifier (HF2TA 50 MHz Current Amplifier, Zurich Instruments AG, Technopark Strasse 1, 8005 Zurich, Switzerland), a *trans*-impedance circuit. The signal is converted to a voltage after passing through the current amplifier. Therefore, in the ESI† figures, voltage is used as the unit for presenting partial results. However, since voltage depends on the amplifier gain, impedance is a more intrinsic and normalized property of the sensors themselves. Consequently, in the main sections, voltage values are converted to impedance, expressed in units of Ω, using the formula below.
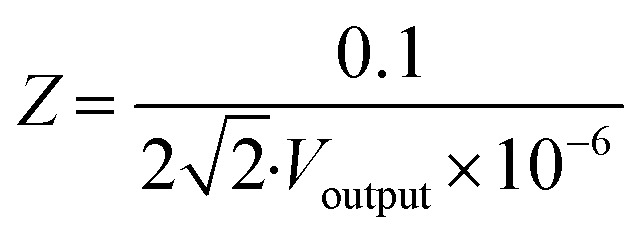


### 2.2 Sensor fabrication

**Fig. 2:**
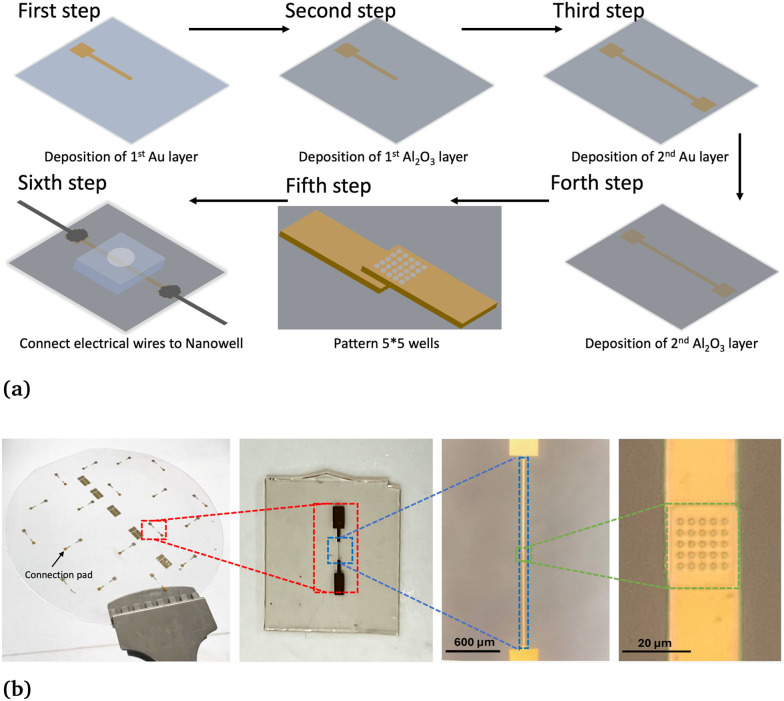
(a) Microfabrication procedures for nanowell sensors. Follow the arrows: first step: first 100 nm of the gold electrode after deposition and lift-off; second step: first 40 nm Al_2_O_3_ layer by ALD; third step: second 100 nm gold electrode using the same fabrication processes as the first layer; fourth step: second 40 nm Al_2_O_3_ layer by ALD; fifth step: zoom-in view of well-shaped arrays in the middle of overlapping area are exposed by multiple etchings for two Al_2_O_3_ layers and one gold layer; sixth step: finish connection setup with conductive wires and epoxy, and install PDMS fluidic cell. (b) From left to right: 1. View of a fabricated wafer; 2. Single nanowell sensor; 3. Microscope view of electrodes; 4. Microscope view of 5 × 5 well-shaped arrays.

A schematic of the key fabrication steps to create the nanowell sensor is presented in Fig. 2a. The sensor is prepared on 7.62 cm-diameter, 500 μm-thick fused silica substrates. The first layer of gold (100 nm) is deposited by physical vapor evaporation (E-beam) on a silica wafer previously covered with a 5 nm chromium layer to improve adhesion. The first electrode is then created from this gold layer by photolithography and lift-off processing (see first step, Fig. 2a). A 40 nm layer of aluminum oxide is subsequently deposited by atomic layer deposition (second step, Fig. 2a). A second 5 nm chromium adhesion is then deposited, followed by a second 100 nm gold layer deposition using the same process as the first layer (third step, Fig. 2a). Note that the first and second layers of gold overlap in a small 20 μm × 20 μm area. Lastly, a 40 nm aluminum oxide layer is deposited as a protection layer on top (fourth step, Fig. 2a). After depositing all metal layers, multiple wet etching steps are performed to pattern the well-shaped array on the overlapping area between the two gold electrodes by coating a layer of photoresist (PR) and etching the two aluminum oxide layers (buffered oxide etchant), one gold layer, and one chromium layer (gold and chromium etchants) to expose the bottom gold layer (see zoom-in view of the array in Fig. 2a, fifth step). After the strip-off of the PR, a second mask is used to feature another layer of PR to etch the aluminum oxide outside the sensor to remove the residue on the silica substrate. A polydimethylsiloxane (PDMS, Sylgard 184, Dow Corning, 10 : 1 prepolymer/curing agent) fluidic cell is glued (5 Minute Epoxy, Devcon, Illinois Tool Works Inc.) above the array of wells to keep the liquids contained in the working area (shown in Fig. 2a, sixth step). To connect the impedance sensor to other electronic equipment, electrically conductive wires are attached to the gold connection pads by conductive epoxy (CW 2400, Chemtronics, Kennesaw, GA, USA), as shown in the last step in Fig. 2a. In Fig. 2b are views of the nanowell array with different magnification, including a view of the wafer after fabrication, a single sensor, the nanowell structure, and the array of wells observed under a bright-field microscope (Ernst Leitz GmbH, Wetzlar, Germany). We used a potentiostat (Gamry Instruments, Warminster, PA, USA) to characterize the impedance spectrum of the biosensor devices, as shown in Fig. S6.†^28^ We performed impedance measurement tests ranging from 10 Hz to 3 MHz on a multitude of fabricated sensors. Beyond 100 kHz, the impedance reaches a constant value and becomes dominated by resistance. Thus, we choose to operate at a frequency of 1 MHz to monitor changes in ionic resistance due to antigen binding.^28^

### 2.3 Preparation of the reagents

Polyclonal and monoclonal SARS-CoV-2 antibodies (R&D Systems, Minneapolis, MN, USA) are used throughout the experiments in a 100 μg mL^−1^ concentration in PBS. No significant differences in results are seen between the type of antibody used, and Fig. S4† shows the overlapping responses of the monoclonal and polyclonal antibodies. The first experiments are performed using the wild-type SARS-CoV-2 spike protein (R&D Systems, Minneapolis, MN, USA) as the target protein and are prepared in artificial human saliva (Sigma-Alrich, St. Louis, MO, USA) in concentrations ranging from 0–1000 ng mL^−1^ (0–7.5 nM). In addition, MERS-CoV spike protein (University of California-San Diego, San Diego, CA, USA) is used to perform a specificity test on a different protein, prepared within 0.18× PBS at a concentration of 1000 ng mL^−1^. The PBS used in this work is 1× (1× PBS, pH = 7.4, Gibco, Thermo Fisher Scientific Inc., Waltham, Massachusetts, US). The diluted PBS used later in this work is diluted by deionized (DI) water from Rutgers Micro Electronics Research Laboratory.

### 2.4 Real-time measurements

Fig. S2† shows the real-time impedance measurement for the first pipetting of (5 μL) 1× PBS to the empty nanowell sensor, monitored under 4 different frequencies. For 1× PBS, following an initial increase caused by pipetting PBS into an empty sensor, which decreases the media's impedance baseline, the impedance subsequently increases, approaching a constant impedance level. The impedance gradually decreases without the existence of any adsorbable material, possibly because of the slight and persistent evaporation inside the sensor. Increasing the frequency results in a higher impedance value. For example, in Fig. S2,† curves from top to bottom represent frequencies from 2 MHz to 100 kHz. 1 MHz is used during the experiments to avoid the parasitic and mutual inductive effect in the system at high frequencies.^26–28^

The experiments presented in this work have the following procedure. First, the sensor is prepared by connecting to the lock-in amplifier and is supplied with a 100 mV, 1 MHz AC signal. The first solution added to the sensor is 5 μL of 1× PBS, and for all steps, the solution resides in the sensor for 10 minutes, undisturbed, before the subsequent step commences. Next, 3 μL more of PBS are added, followed by 3 μL of antibodies prepared in PBS that are adsorbed to the surface of the sensor. The power source is then removed, and the 11 μL of liquid is removed from the sensor *via* a pipette. After the liquid is fully removed, the power is restored, and two more rounds of PBS are added, as mentioned above, to recreate the liquid environment. Lastly, 3 μL of SARS-CoV-2 spike protein prepared in artificial saliva of the target concentration is added to the sensor.

**Fig. 3:**
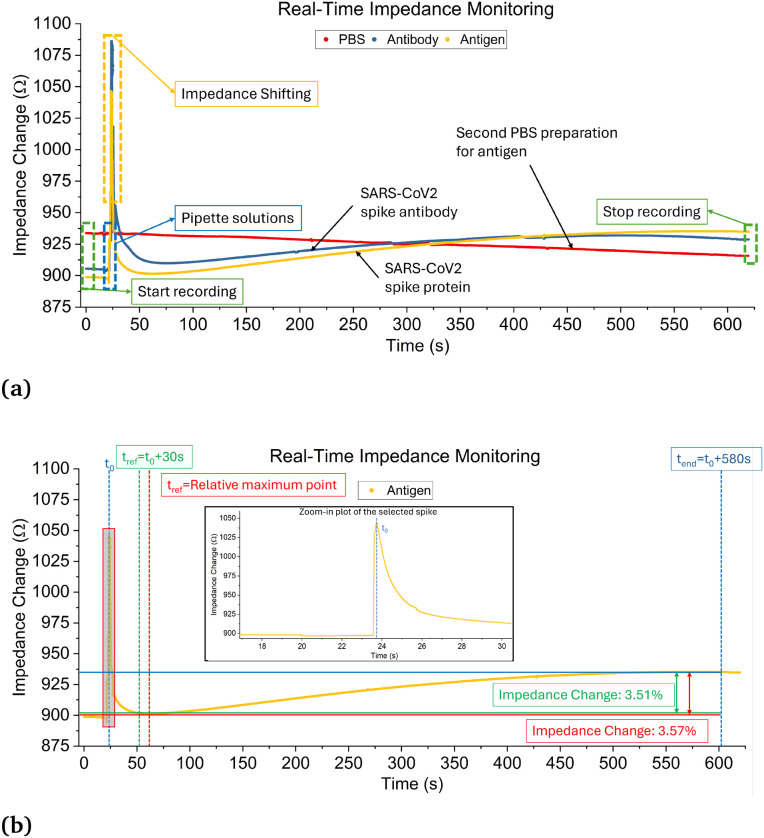
An example of the data analysis methods using 1000 ng mL^−1^ SARS-CoV-2 spike protein samples. (a) Comparison of real-time impedance monitoring after pipetting 1× PBS, antibodies, and antigens. (b) Comparison of two analysis methods.

Fig. 3a is a plot of the recorded impedance. Shown in red is the impedance response of the first round of 3 μL of PBS added to the sensor, blue is the addition of the antibody solution, and yellow is 1000 ng mL^−1^ antigen suspended in artificial saliva. Before the real-time test, the sensor is prepared with antibodies. In this figure, the blue curve represents the impedance change during antibody injection. As the curve descends, it indicates that the antibodies are binding to the sensor, which causes an increase in impedance. When the liquid solutions are added, inserting the pipette tip into the well causes large shifts in impedance before stabilizing over a short period of time. The red PBS curve displays a slowly decreasing impedance due to the enhanced conductivity provided by the PBS. The addition of the antibodies increases impedance due to their adsorption to the sensor surface and demonstrates that the sensor is functional. Fig. 3b is an isolated view of the yellow spike protein response curve. Due to every experiment having slightly different pipette insertion times after the recording is started, three-time instances: *t*_0_, *t*_ref_, and *t*_end_ are used to calculate the increase in impedance for each trial and will be used to evaluate the performance of the sensor. *t*_0_ is the location of the last shift in impedance due to pipette insertion, *t*_ref_ is 30 s after *t*_0_, and tend is 580 s after *t*_0_. The increase in impedance is then calculated as (*Z*_*t*_ref__ − *Z*_*t*_end__)/*Z*_*t*_ref__. A previous method to calculate the impedance change is to instead use the time of the relative maximum after *t*_0_ as *t*_ref_.^25,27,28^ Both methods will be used and compared in this study. Using the first method results in an impedance increase of 3.51%, shown in green, and the second is 3.57%, shown in red. The new method was developed due to some signals not having a clearly defined relative maximum. Thus, the new method is a more robust approach to interpreting the results.

## 3 Results

### 3.1 SARS-CoV-2 spike protein responses in artificial saliva

**Fig. 4:**
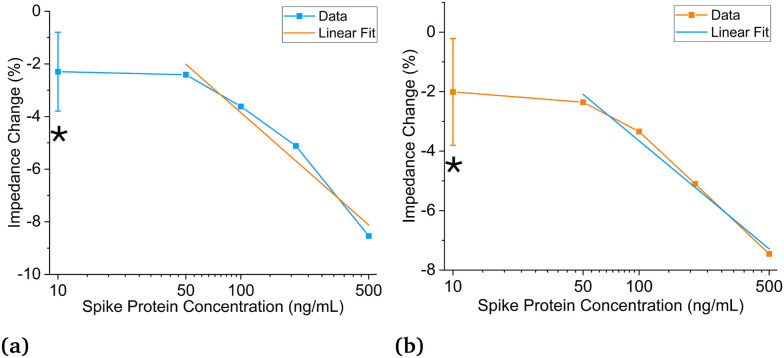
Sensing of spike protein in saliva with antibody in 1× PBS solution. (a) Titration curve using updated data analysis method for SARS-CoV-2 spike proteins in saliva and antibodies in 1× PBS with 50–500 ng mL^−1^ dynamic range and 200 ng mL^−1^ (1.5 nM) detection limit; linear fit: (*Z*) = 8.36–6.11 × (C), *R*-square = 0.97. (b) Titration curve using previous data analysis method for SARS-CoV-2 spike proteins in saliva and antibodies in 1× PBS with 50–500 ng mL^−1^ dynamic range and 200 ng mL^−1^ (1.5 nM) detection limit; linear fit: (*Z*) = 6.74–5.2 × (C), *R*-square = 0.99, * Denotes negative control (*C* = 0 ng mL^−1^) plotted at *C* = 10 ng mL^−1^ for visualization purposes, *N* = 5 for *C* = 0 ng mL^−1^, *N* = 1 for all other concentrations.

Experiments are performed using antibodies in 1× and 0.18× PBS solutions paired with SARS-CoV-2 spike proteins suspended in artificial saliva. Multiple sensors are used during these experiments. The sensors are fragile and typically do not survive more than two to three experiments before having to be discarded. The first experiments used antibodies in 1× PBS solutions. Fig. 4a shows the data analysis results based on the first method described above, while Fig. 4b demonstrates the analysis results using the second method.^25,27,28^ From these two figures, the negative control (NC) (*C* = 0 ng mL^−1^) overlaps with 100–200 ng mL^−1^. Thus, the LOD is estimated to be not less than 200 ng mL^−1^ (1.5 nM, molar concentration = (mass of solute/volume of solution) × (1/molecular weight) where the molecular weight is 134 kDa for SARS-CoV-2 spike protein) which is not ideal and could be due to the differences in impedance between the 1× PBS and artificial saliva.

As we speculate the impedance difference between 1× PBS solution and saliva may negatively affect detection sensitivity, we next seek to use a PBS solution that has matching impedance. Fig. S3a† shows the voltage responses for five different concentrations of PBS, ranging from 0.1× to 1×, and artificial saliva. In Fig. S3b,† the output voltage for artificial saliva is between 0.1× and 0.2× PBS. A regression curve is then plotted for the different PBS solutions to find the equivalent PBS concentration for saliva. The output voltages at 300 seconds are used for all tests, and the equivalent PBS concentration for saliva is found to be 0.18× PBS through interpolation.

**Fig. 5:**
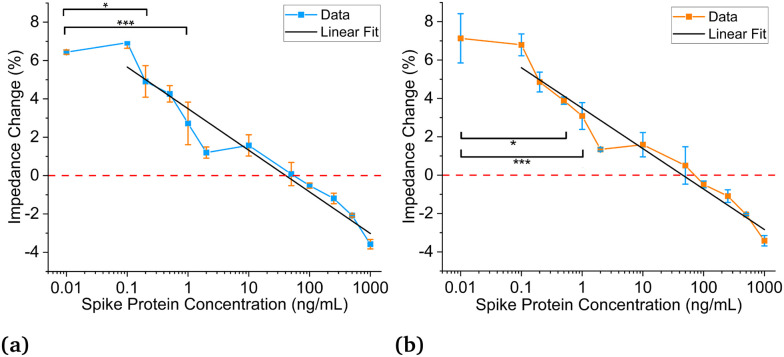
Sensing of spike protein in saliva with antibody in 0.18× PBS solution. Negative control (*C* = 0 ng mL^−1^) plotted at *C* = 0.01 ng mL^−1^ for visualization purposes. (a) Data using updated data analysis method for SARS-CoV-2 spike proteins in saliva and antibodies in 0.18× PBS with 0.1–1000 ng mL^−1^ dynamic range; linear fit: (*Z*) = 3.49–2.17 × log (C), *R*-square = 0.94. (b) Data using previous data analysis method for SARS-CoV-2 spike proteins in saliva and antibodies in 0.18× PBS with 0.1–1000 ng mL^−1^ dynamic range; linear fit: (*Z*) = 3.49–2.11 × log (C), *R*-square = 0.95. *P ≤ 0.05; ***P ≤ 0.0001, *N* = 2 for *C* = 0 ng mL^−1^, *N* = 5 for *C* = 50 ng mL^−1^, *N* = 3 for all other concentrations.

Fig. 5a and b show the titration curves for antibodies suspended in 0.18× PBS buffer to match the baseline of saliva. Fig. 5a uses the new data analysis method, and Fig. 5b uses the previous method. For these results, concentrations were titrated from 0.1–1000 ng mL^−1^. Fig. 5a displays a statistically significant difference in impedance response between 0 and 0.2 ng mL^−1^ (1.5 pM) using a 0.05 significance level, which is a three-order-of-magnitude improvement compared to the estimated limit of detection using 1× PBS. When using an even higher significance level of 0.0001, the detection limit is 1 ng mL^−1^ (7.5 pM) and is still substantially higher than the previous estimate. Fig. 5b displays a 0.5 ng mL^−1^ (3.75 pM) detection limit using a 0.05 significance level, slightly worse than the new analysis method. However, when using a significance level of 0.0001, it also has a 1 ng mL^−1^ (7.5 pM) detection limit. The detection limits for significance levels from 0.05 to 0.0001 can be seen in Table S1.† The linear fits can be seen in both plots, and the intersection of the fits and the negative controls results in theoretical detection limits of 0.13 ng mL^−1^ (0.97 5 pM) and 0.33 ng mL^−1^ (2.475 pM) for the new and previous methods, respectively.

The LODs for antibodies prepared in 0.18× PBS solutions are much lower than those in 1× PBS solutions. All titration curves in Fig. 4 and 5 show similar and strong linear relationships; however, Fig. 5 displays much lower LODs than Fig. 4. Using 0.18× PBS, the new and old analysis methods result in experimental LODs of 0.2 (1.5 pM) and 1 ng mL^−1^ (7.5 pM) respectively, compared to 200 ng mL^−1^ (1.5 nM) with the 1× PBS solution. Thus, 0.18× PBS is a more suitable buffer for SARS-CoV-2 spike antibodies for this application. Lastly, the agreement between the two data analysis methods for the antibodies prepared in 1× and 0.18× PBS gives credence to the matched baseline voltage having a significant impact on the LOD and is not artificially lowered by the data analysis method employed.

### 3.2 MERS-CoV specificity tests

After demonstrating that the sensor can detect a binding event between SARS-CoV-2 spike protein and a matching antibody, the specificity of the sensor is examined by applying MERS-CoV spike proteins to a sensor prepared with SARS-CoV-2 antibodies. MERS-CoV is used for the specificity test as it is a coronavirus closely related to SARS-CoV-2.^31^ In these experiments, MERSCoV spike protein with a concentration of 1000 ng mL^−1^ in 0.18× PBS is introduced to the sensor using the same procedure as before. In previous experiments, there are no obvious differences between using artificial saliva and 0.18× PBS as protein buffers. A spike protein concentration of 100 ng mL^−1^ prepared in artificial saliva and 0.18× PBS had voltage drops of 0.49 and 0.64% respectively, as shown in Fig. S5,† which were within uncertainty bounds. Therefore, the use of a 0.18× PBS buffer is not expected to affect the specificity tests for MERS-CoV spike protein in this section.

**Fig. 6:**
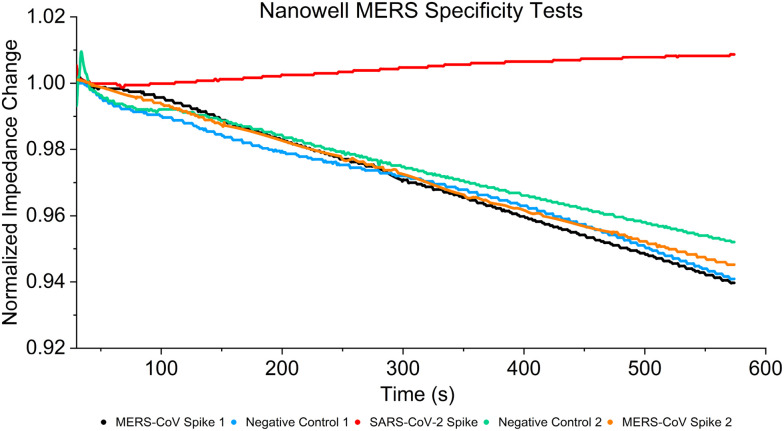
Comparisons between MERS-CoV spike proteins, SARS-CoV-2 spike proteins, and negative control using SARS-CoV-2 spike antibodies. Only the red SARS-CoV-2 spike protein curve gives a response to the SARS-CoV-2 antibodies, and MERS-CoV curves follow the same trends as the negative control curves, proving the device is capable of differentiating SARS-CoV-2 proteins from similar respiratory disease samples.

Fig. 6 shows the results of the specificity tests and displays that the sensor can differentiate between the two spike proteins. Two negative controls (negative control 1 and negative control 2 in Fig. 5) tests are shown in blue and green, two MERS-CoV spike protein curves result in black and orange, and a SARS-CoV-2 spike protein results in red (250 ng mL^−1^). All curves are normalized at 50 seconds to facilitate comparison by accounting for variations in impedance baselines across different sensors, thereby reducing the difficulty of observing all five curves. The MERS-CoV spike protein responses overlap with the negative controls, demonstrating that the sensor does not detect a binding event between the MERS-CoV spike protein and SARS-CoV-2 antibody and instead decreases in impedance due to the presence of the PBS buffer, thus displaying the ability of the sensor to differentiate between the two antigens.

## Discussion

Table 1 compares different biosensors for COVID-19 detection and highlights the key differences in their detection capabilities, sensitivity, and practical applications. The nanowell-based impedance sensor demonstrates a strong balance between sensitivity, speed, and ease of use, making it a highly competitive alternative among existing technologies. With a limit of detection (LOD) of 0.2 ng mL^−1^ (1.5 pM), it offers real-time impedance-based detection, requiring only a small sample volume (<5 μL) and delivering results within 10 minutes. Its portability and low sample preparation requirements make it particularly suitable for point-of-care (PoC) diagnostics.

**Table 1:** Comparison of different biosensors for COVID-19 detection

Biosensor type	LOD (ng mL^−1^)	Sensitivity	Measurement range (ng mL^−1^)	Sample volume (μL)	Time (min)	Portability	Sample preparation	Ref.
Paper-based biosensors	0.1–10	Moderate-high	1–1000	10–50	15–30	Very high	Low	Pinheiro *et al.*^32^
Electrochemical biosensors	0.01–10	High	0.1–1000	5–50	10–30	High	Medium	Patel *et al.*^33^
FET biosensors	0.000001–1	Very high	0.000001–1000	1–10	<10	High	Medium	Wasfi *et al.*^34^
Optical biosensors	0.01–1	Very high	0.01–1000	5–100	5–20	Medium	Medium	Irkham *et al.*^35^
Nanomaterial-based biosensors	0.01–10	Very high	0.01–500	5–50	10–30	Medium-high	Medium	Truong *et al.*^36^
Nanowell-based impedance sensor (this work)	0.2 (1.5 pM)	High	0.1–1000	<5	10	High	Low	This work

Paper-based biosensors are among the most portable and cost-effective solutions, requiring minimal sample preparation and simple handling procedures.^32^ However, their LOD typically ranges from 0.1 to 10 ng mL^−1^, which is higher compared to other biosensing technologies. They offer moderate-to-high sensitivity but often require longer processing times (15–30 minutes), making them less efficient for rapid diagnostics. Despite these limitations, their ease of use and affordability make them ideal for mass screening applications in low-resource settings.^32^

Electrochemical biosensors, on the other hand, provide higher sensitivity and a broader detection range (0.1–1000 ng mL^−1^), making them more adaptable for different sample concentrations.^33^ These biosensors generally require 5–50 μL of sample volume and can deliver results within 10–30 minutes. While they offer good portability, electrode functionalization adds complexity to sample preparation, which may limit their practical implementation in non-laboratory environments.^33^

Field-effect transistor (FET) biosensors are among the most ultrasensitive COVID-19 detection platforms, with an LOD as low as 1 fg mL^−1^, making them highly suitable for early-stage virus detection.^34^ These devices are designed for real-time detection with rapid response times (<10 minutes). However, FET biosensors require complex fabrication processes, making them less accessible for large-scale deployment despite their remarkable sensitivity.^34^

Optical biosensors, including surface plasmon resonance (SPR) and lab-on-a-chip platforms, offer high specificity and real-time detection capabilities, with an LOD typically ranging from 0.01 to 1 ng mL^−1^.^35^ They can process sample volumes between 5–100 μL, generating results within 5–20 minutes. While they provide excellent sensitivity, their portability is limited due to the need for optical alignment and specialized benchtop equipment.^35^

Nanomaterial-based biosensors leverage advanced nanotechnology to enhance sensitivity and specificity for COVID-19 detection, with an LOD range of 0.01–10 ng mL^−1^.^36^ These biosensors often require moderate sample preparation and exhibit a measurement range of 0.01–500 ng mL^−1^. Their moderate-to-high portability makes them appealing for PoC applications; however, their fabrication can be technically demanding due to the synthesis and functionalization of nanomaterials.^36^

Among these biosensing technologies, the nanowell-based impedance sensor offers a promising balance of speed, sensitivity, and user-friendliness. Unlike many traditional biosensors that require extensive sample preparation or complex instrumentation, the platform achieves rapid detection (10 minutes) with minimal sample volume (5 μL), making it particularly suited for point-of-care and field-deployable applications. These attributes position it as a highly competitive alternative to existing biosensors, particularly in clinical and resource-limited settings.

## Conclusions

A label-free and rapid microfabricated impedance biosensor is presented that can detect SARS-CoV-2 spike protein successfully. SARS-CoV-2 spike proteins in saliva with antibodies in 1× PBS displayed an experimental limit of detection of 200 ng mL^−1^ (1.5 nM) within 10 minutes. To lower the LOD, the baseline voltage output of different concentrations of PBS was investigated to find the best match for the artificial saliva. Through a regression curve, 0.18× PBS was the resultant match, and the experimental LOD with this concentration of PBS was lowered to 0.2 ng mL^−1^ (1.5 pM). Apart from the comparison between different PBS buffers, the data analysis method from previous works^25,27–29,37,38^ was updated to remove the need for a clearly defined relative maximum. Both methods provide similar results, with the new method having slightly lower LODs, but the agreement between the approaches confirms the benefit of matching the baseline conductivity. In the following section, the sensor specificity is investigated by measuring the impedances of the MERS-CoV spike, SARS-CoV-2 spike, and negative control (pure PBS buffer). The responses of the MERS-CoV spike proteins mimic the negative controls and demonstrate that the impedance sensor can differentiate between the target proteins and non-related samples. Comparing the performance of the nanowell sensor to others in the literature, the sensor has a strong balance of speed, sensitivity, and user-friendliness that is not seen among others. These experiments successfully verify the feasibility of using this nanowell sensor as a strong candidate to detect SARS-CoV-2 in human samples. This paper presents the possibility of using our nanowell biosensors to detect the SARS-CoV-2 spike proteins. In the future stages, we will utilize the whole virus or pseudovirus to promote the SARS-CoV-2 detection in the real world.

## Author contributions

Zhuolun Meng: methodology, formal analysis, investigation, data curation, writing – original draft, writing – review & editing, software, validation, visualization. Liam White: methodology, formal analysis, investigation, data curation, writing – original draft, writing – review & editing, software, validation, visualization. Pengfei Xie: methodology, formal analysis, investigation, data curation, validation, visualization, writing – review & editing. Hassan Raji: methodology, formal analysis. S. Reza Mahmoodi: methodology, formal analysis, investigation, data curation, validation, visualization, writing – review & editing. Aris Karapiperis: formal analysis, investigation, data curation, writing – review & editing. Hao Lin: conceptualization, methodology, funding acquisition, project administration, supervision, writing – review & editing. German Drazer: conceptualization, methodology, funding acquisition, project administration, supervision, writing – review & editing. Mehdi Javanmard: conceptualization, methodology, funding acquisition, project administration, supervision, writing – review & editing. Edward P. DeMauro: conceptualization, methodology, funding acquisition, project administration, supervision, writing – review & editing.

## Conflicts of interest

There are no conflicts to declare.

## Supplementary Material

SD-004-D5SD00002E-s001

SD-004-D5SD00002E-s002

## Data Availability

The data supporting this study's findings are available in the ESI.† Other data are subject to the following conditions: available upon reasonable request. For additional information, please get in touch with the corresponding author.
