# Supplementary figures and images for: A label-free nanowell-based impedance sensor for ten-minute SARS-CoV-2 detection
Source: Sens Diagn. 2025 Apr 30;4(6):511–8. doi: 10.1039/d5sd00002e (PMC12056702; doi:10.1039/d5sd00002e)

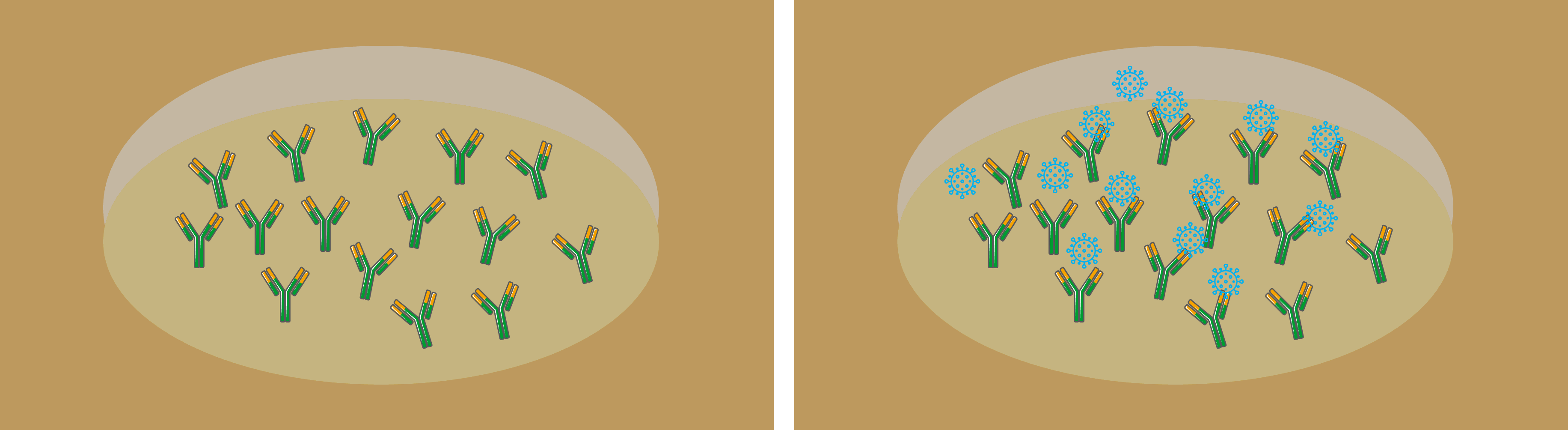

Supplement: SD-004-D5SD00002E-s001 [file SD-004-D5SD00002E-s001.zip › ESI figures/FigS1.png]

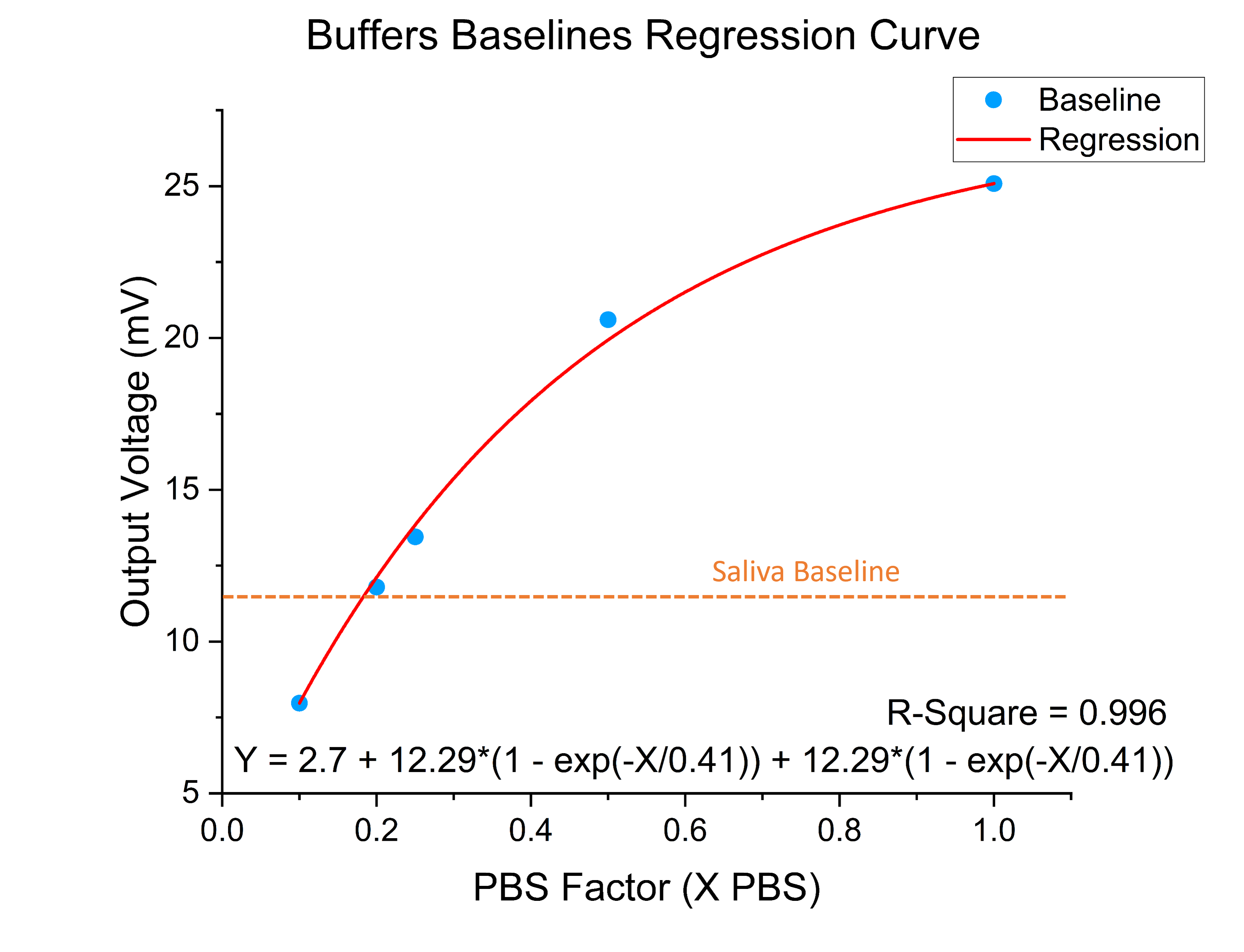

Supplement: SD-004-D5SD00002E-s001 [file SD-004-D5SD00002E-s001.zip › ESI figures/FigS3b.png]

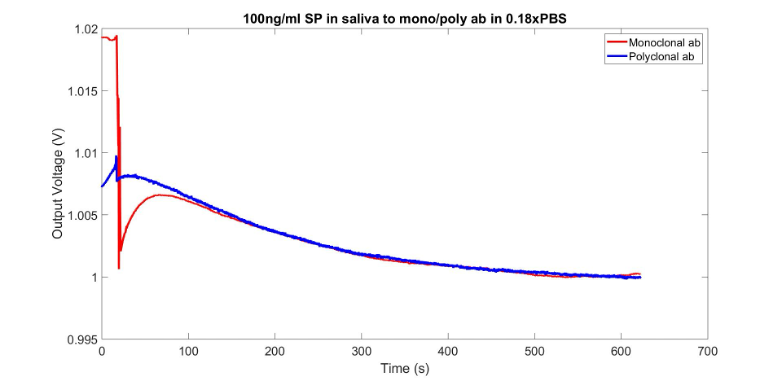

Supplement: SD-004-D5SD00002E-s001 [file SD-004-D5SD00002E-s001.zip › ESI figures/FigS4.png]

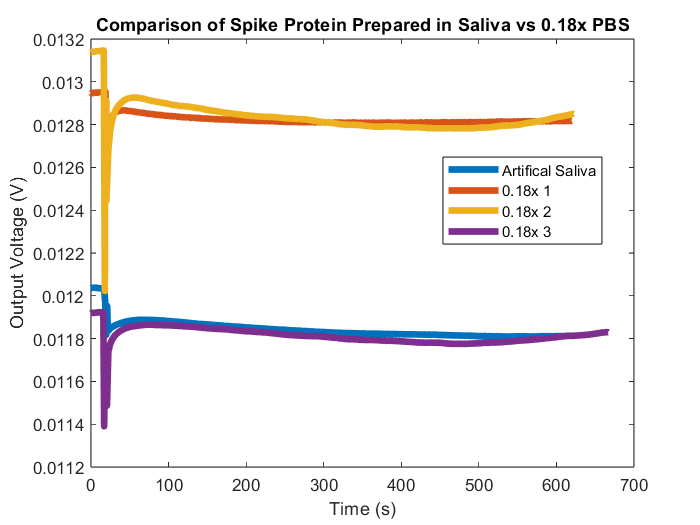

Supplement: SD-004-D5SD00002E-s001 [file SD-004-D5SD00002E-s001.zip › ESI figures/FigS6.png]

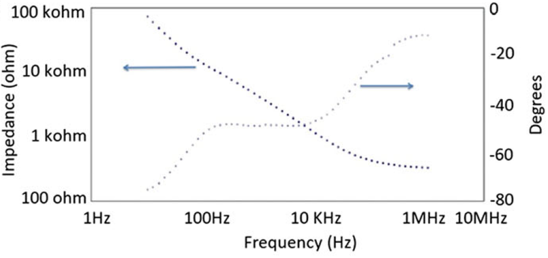

Supplement: SD-004-D5SD00002E-s001 [file SD-004-D5SD00002E-s001.zip › ESI figures/FigS7a.png]

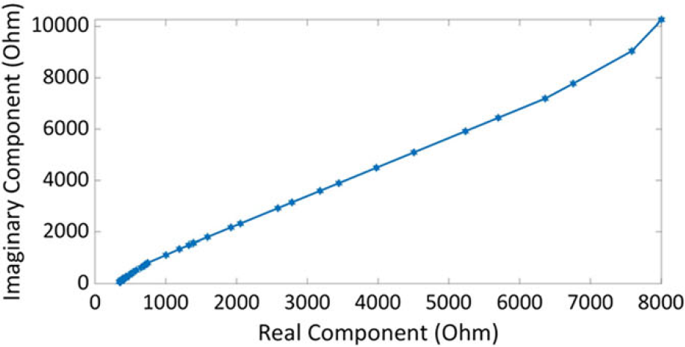

Supplement: SD-004-D5SD00002E-s001 [file SD-004-D5SD00002E-s001.zip › ESI figures/FigS7b.png]
