# Supplementary material for: A label-free nanowell-based impedance sensor for ten-minute SARS-CoV-2 detection
Source: Sens Diagn. 2025 Apr 30;4(6):511–8. doi: 10.1039/d5sd00002e (PMC12056702; doi:10.1039/d5sd00002e)
Supplement: SD-004-D5SD00002E-s002 [file SD-004-D5SD00002E-s002.pdf]

Supplementary Information (SI) for Sensors & Diagnostics.
This journal is © The Royal Society of Chemistry 2025

Supplementary

Zhuolun Meng,[†,‡] Liam White,[¶,‡] Pengfei Xie,[†] Hassan Raji,[†] S. Reza Mahmoodi,[†] Aris Karapiperis,[¶] Hao Lin,[¶] German Drazer,[¶] Mehdi Javanmard,[*,†] and Edward P. DeMauro[*,¶]

†*Department of Electrical and Computer Engineering, Rutgers University-New Brunswick, Piscataway, NJ, USA, 08854*

‡*These authors contributed equally to this work*

¶*Department of Mechanical and Aerospace Engineering, Rutgers University-New Brunswick, Piscataway, NJ, USA, 08854*

E-mail: mehdi.javanmard@rutgers.edu; edward.demauro@rutgers.edu

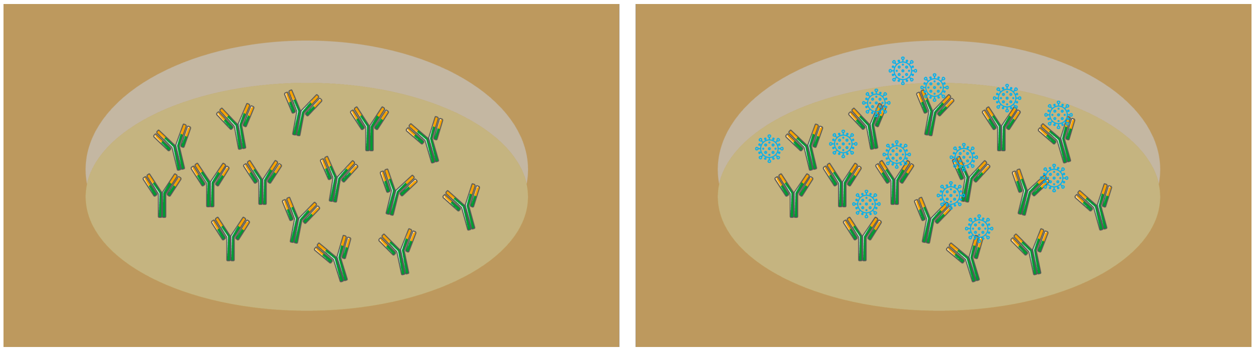

Figure S1: Top view of a single nanowell adsorbing antibodies and adsorbing target proteins

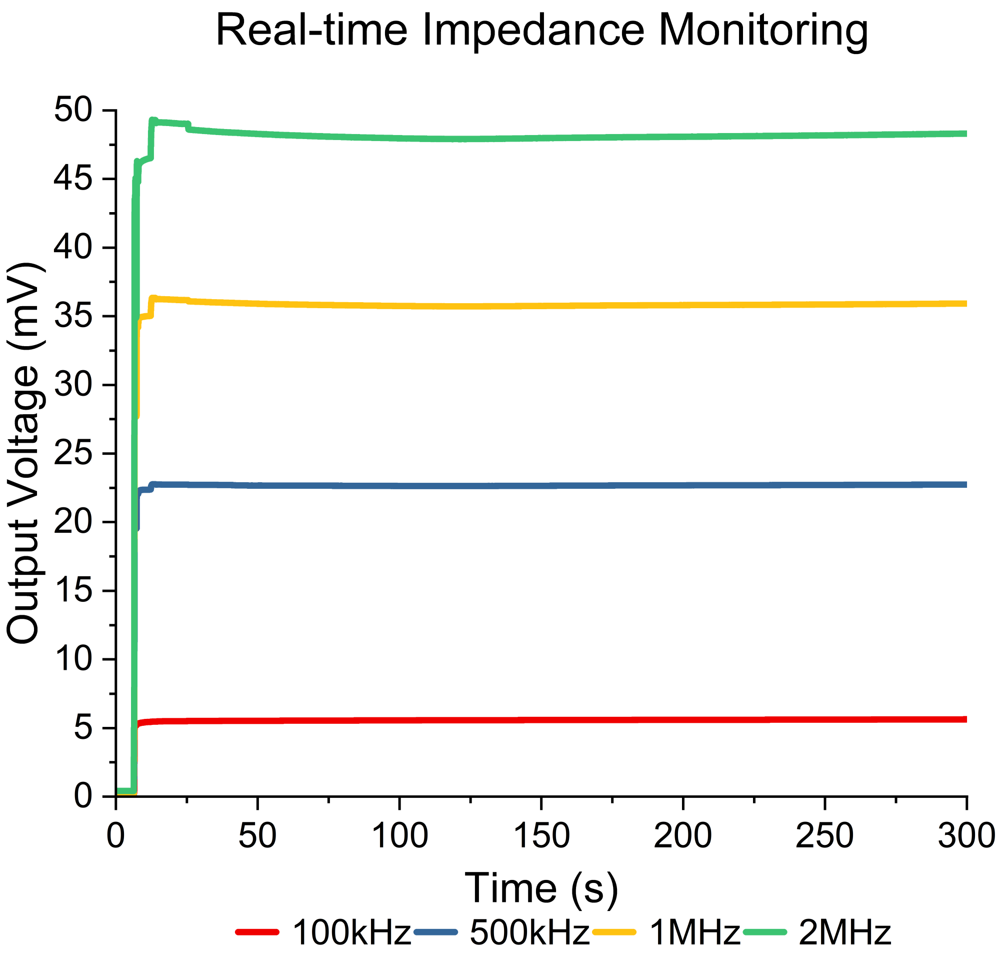

Figure S2: Real-time impedance monitoring four different frequencies under the liquid environment of 1X PBS

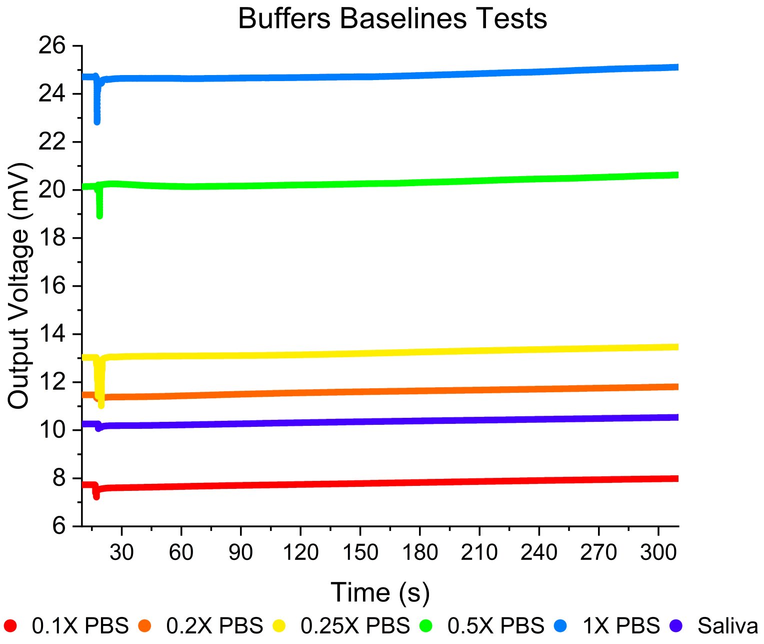

(a)

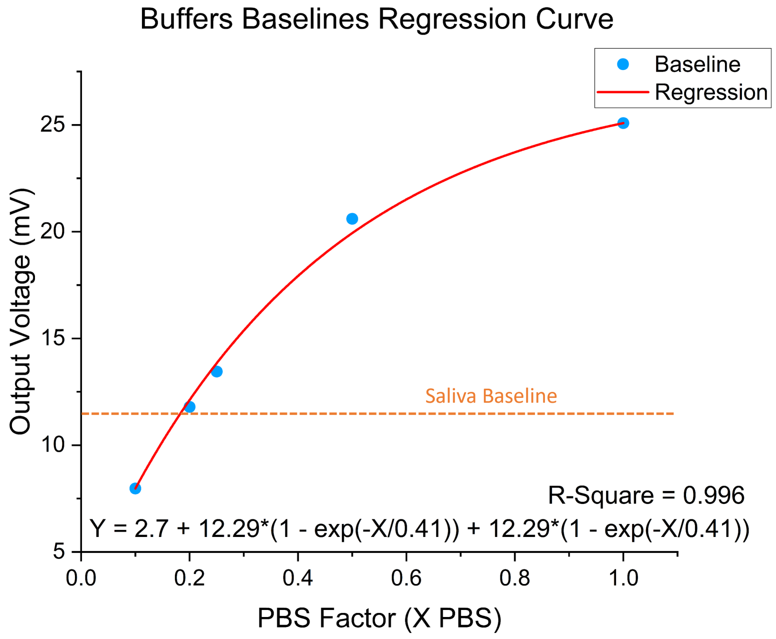

(b)

Figure S3: (a) Output voltage baselines for different concentrations of PBS and saliva. (b) Regression curve for different concentrations of PBS. Multiple experiments with different concentrations of PBS and saliva were performed, and the corresponding PBS concentration of saliva from the regression model is 0.18X PBS.

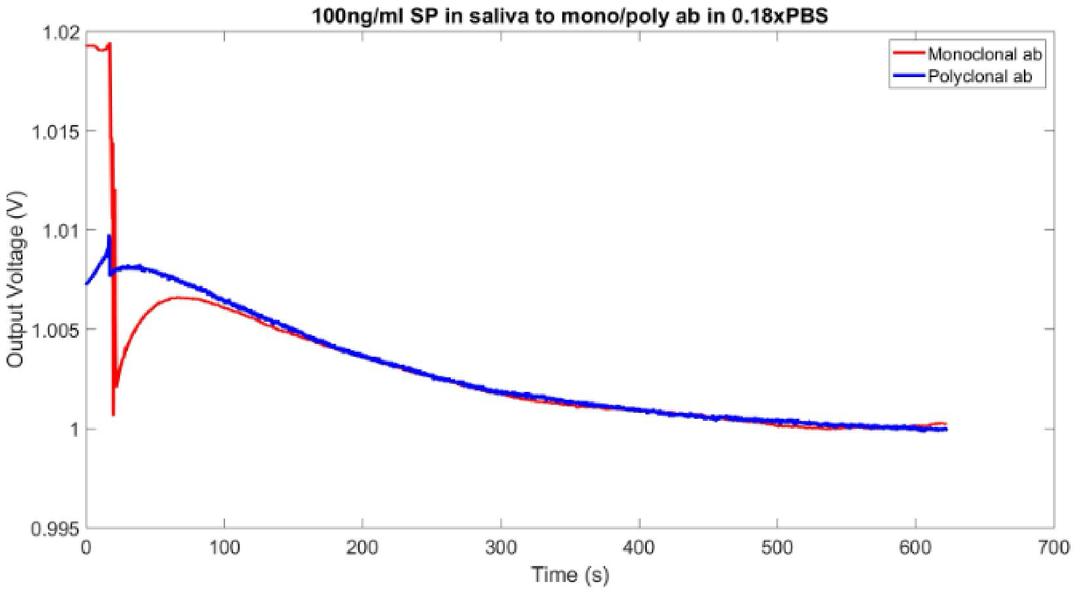

Figure S4: Comparison between Monoclonal and Polyclonal antibodies. The two curves have similar shapes and overlap around 150 seconds, which demonstrates that there appears to be no difference between Monoclonal and Polyclonal antibodies.

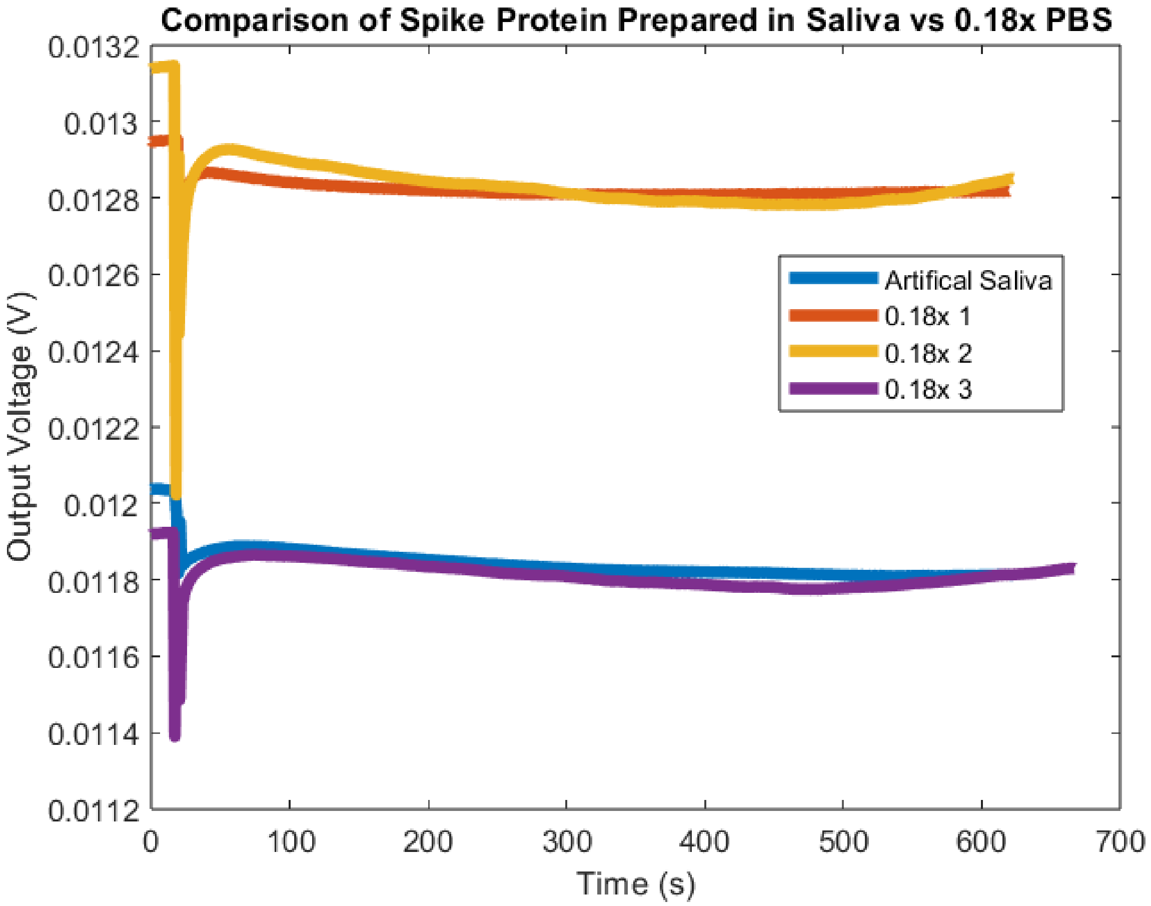

Figure S5: Comparison between SARS-CoV-2 spike protein antigen prepared in artificial saliva and 0.18X PBS. The antigen prepared in artificial saliva, shown in blue, has a similar output to the antigens prepared in 0.18X PBS. The voltage drop for spike protein prepared in artificial saliva is 0.49%, which is within the error bars for 100 ng/mL of spike protein prepared in 0.18X PBS.

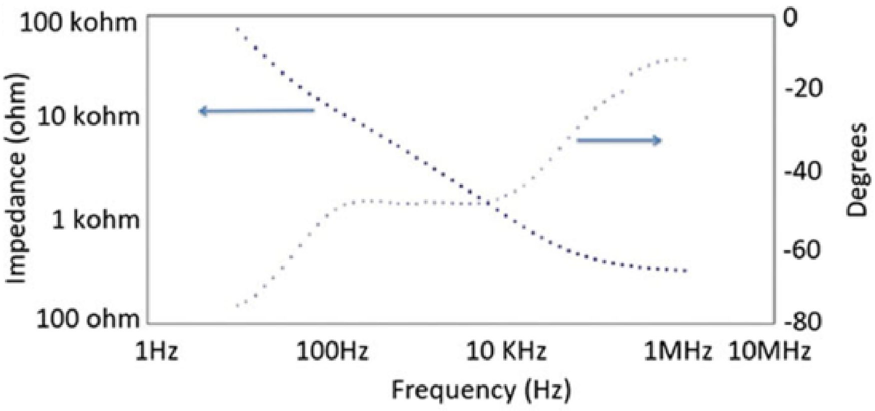

(a)

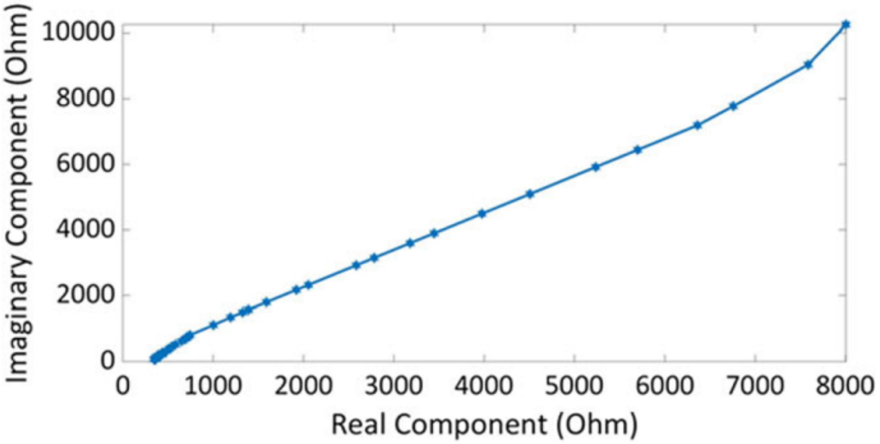

(b)

Figure S6: (a) Magnitude and Phase of Impedance spectrum of the device.[1] (b) The Nyquist curve of the corresponding device. The device starts out capacitive at low frequencies, and response becomes dominated by solution resistance as frequency is increased beyond 100 kHz. At 1 MHz, the response is resistive, which is the optimal operation regime for the sensor. Accumulation of protein in micro-well results in an increase in resistance between electrodes.[1]

Table S1: Table of detection limits for new and old data analysis methods using spike protein suspended in artificial saliva and antibodies suspended in 0.18X PBS for confidence levels ranging from 0.0001 - 0.05.

**Analysis Method**	**Limit of Detection (ng/mL)**	**Limit of Detection (pM)**	**P**
New	0.2	1.5	≤ 0.05
-	1.0	7.5	≤ 0.001
-	1.0	7.5	≤ 0.0001
Old	0.5	3.7	≤ 0.05
-	1.0	7.5	≤ 0.001
-	1.0	7.5	≤ 0.0001

References

(1) Xie, P.; Song, N.; Shen, W.; Allen, M.; Javanmard, M. A ten-minute, single step, label-free, sample-to-answer assay for qualitative detection of cytokines in serum at femtomolar levels. *Biomedical Microdevices* **2020**, *22*, 1–9.
